# Structural determinants of alternating (α1 → 4) and (α1 → 6) linkage specificity in reuteransucrase of *Lactobacillus reuteri*

**DOI:** 10.1038/srep35261

**Published:** 2016-10-17

**Authors:** Xiangfeng Meng, Tjaard Pijning, Justyna M. Dobruchowska, Huifang Yin, Gerrit J. Gerwig, Lubbert Dijkhuizen

**Affiliations:** 1 Microbial Physiology, Groningen Biomolecular Sciences and Biotechnology Institute (GBB), University of Groningen, Nijenborgh 7, 9747 AG Groningen, The Netherlands; 2 Biophysical Chemistry, Groningen Biomolecular Sciences and Biotechnology Institute (GBB), University of Groningen, Nijenborgh 7, 9747 AG Groningen, The Netherlands

## Abstract

The glucansucrase GTFA of *Lactobacillus reuteri* 121 produces an α-glucan (reuteran) with a large amount of alternating (α1 → 4) and (α1 → 6) linkages. The mechanism of alternating linkage formation by this reuteransucrase has remained unclear. GTFO of the probiotic bacterium *Lactobacillus reuteri* ATCC 55730 shows a high sequence similarity (80%) with GTFA of *L. reuteri* 121; it also synthesizes an α-glucan with (α1 → 4) and (α1 → 6) linkages, but with a clearly different ratio compared to GTFA. In the present study, we show that residues in loop977 (^970^DGKGYKGA^977^) and helix α4 (^1083^VSLKGA^1088^) are main determinants for the linkage specificity difference between GTFO and GTFA, and hence are important for the synthesis of alternating (α1 → 4) and (α1 → 6) linkages in GTFA. More remote acceptor substrate binding sites (i.e.+3) are also involved in the determination of alternating linkage synthesis, as shown by structural analysis of the oligosaccharides produced using panose and maltotriose as acceptor substrate. Our data show that the amino acid residues at acceptor substrate binding sites (+1, +2, +3…) together form a distinct physicochemical micro-environment that determines the alternating (α1 → 4) and (α1 → 6) linkages synthesis in GTFA.

Lactic acid bacteria (LAB) have been widely explored for the production of fermented food, due to their ability of producing lactic acid and their generally recognized as safe (GRAS) status^1^. Another interesting property of LAB is their capability of synthesizing exopolysaccharides, which have been used as thickening, texturizing and gelling agents in the food industry^2^,^3^,^4^,^5^,^6^,^7^. Moreover, some of these exopolysaccharides have also been shown to possess prebiotic properties and hold potential to improve gut health^2^,^4^,^7^. Glucansucrases of LAB are responsible for converting sucrose into α-glucans, one of the homopolysaccharides produced by LAB^5^,^8^,^9^,^10^,^11^. Belonging to glycoside hydrolase family 70 (GH70), glucansucrase enzymes contain an (β/α)_8_ barrel structure in their catalytic domain similar to the closely related GH13 family enzymes^8^,^9^,^10^,^11^,^12^,^13^. However, unlike the GH13 enzymes, the (β/α)_8_ barrel structure of glucansucrase enzymes is circularly permuted^14^. The four conserved regions, which have been identified in GH13 family enzymes, are also found in GH70 glucansucrase enzymes^8^,^9^,^10^,^11^,^15^. Due to the circularly permuted arrangement, region I of glucansucrases is located C-terminal to regions II, III and IV (Fig. 1a). Glucansucrases employ a double displacement mechanism to catalyze their reactions^8^,^9^,^10^,^16^. In the first step, the glycosidic linkage of sucrose is cleaved with the formation of a β-glucosyl-enzyme intermediate. In the second step, the glucosyl moiety is transferred to an acceptor substrate, with the retention of α-anomeric configuration. Depending on the available acceptor substrates, glucansucrase enzymes catalyze three different reactions^8^,^9^,^10^,^11^. Polysaccharides are synthesized from sucrose when growing glucan chains are used as acceptor substrate. When short chain oligosaccharide acceptors (e.g. maltose and isomaltose) are available, oligosaccharides are produced at the expense of polysaccharide synthesis. Water also can act as acceptor substrate, resulting in hydrolysis of sucrose into glucose and fructose.

Different glucansucrase enzymes produce α-glucans with a vast structural diversity, differing in linkage composition, size and branching degree^8^,^9^,^10^,^11^, resulting in a wide range of physico-chemical properties and offering opportunities in various applications^5^,^6^,^11^. The most studied α-glucan is dextran, which mainly contains (α1 → 6) linkages and has different degrees of branching (α1 → 3) linkages^5^,^6^,^11^. Dextran produced by *Leuconostoc mesenteroides* NRRL B-512F is produced on an industrial scale and is used as separation matrix in biotechnology, as blood plasma expander in medicine and as food ingredient in food industry^6^. On the other hand, most of the α-glucans produced by *Streptococcus* strains are found to predominantly contain (α1 → 3) linkages, and are named mutans; they are generally insoluble and have been recognized as an important pathogenic factor in the development of dental caries^5^,^8^,^9^,^10^,^11^,^17^,^18^,^19^,^20^. *L. mesenteroides* NRRL B-1355 produces a special glucansucrase ASR, which synthesizes alternan with alternating (α1 → 6) and (α1 → 3) linkages^21^,^22^, and is incapable of synthesizing two consecutive (α1 → 3) linkages^23^. The alternation of (α1 → 6) and (α1 → 3) linkages in alternan makes this polysaccharide resistant to endodextranase hydrolysis and is considered to be the determinant for its distinct physical properties^24^. Therefore, ASR has been explored to produce novel oligosaccharides, containing different linkages from that of dextransucrase, by adding a variety of exogenous acceptor substrates^23^,^25^,^26^,^27^,^28^. These alternansucrase-derived oligosaccharides displayed prebiotic activities in *in vitro* tests^29^,^30^,^31^,^32^. However, the mechanism of alternating linkage formation has remained largely unknown.

*Lactobacillus*, another genus of LAB, also produces α-glucans using glucansucrase enzymes^33^. *Lactobacillus reuteri* 121 produces an α-glucan with a large amount of (α1 → 4) linkages (58%) besides (α1 → 6) linkages (42%), named as reuteran^34^,^35^,^36^. GTFA of *L. reuteri* 121, which is responsible for the synthesis of reuteran, shared the highest similarity (59%) with ASR of *L. mesenteroides* NRRL B-1355 at the time of its discovery^35^. The structure of the reuteran polysaccharide produced by GTFA was investigated in our previous study and a composite model was constructed (Fig. 2)^34^. It contains a large amount of alternating (α1 → 4) and (α1 → 6) linkages; no consecutive (α1 → 6) linkages were detected. Later on, the oligosaccharides produced by GTFA also were isolated and structurally characterized^37^. Incubation of GTFA with sucrose resulted in the synthesis of linear oligosaccharides with alternating (α1 → 4) and (α1 → 6) linkages. This is similar to ASR of *L. mesenteroides* NRRL B-1355, which produces alternating (α1 → 3) and (α1 → 6) linkages. Whether the two enzymes use a similar mechanism to synthesize alternating glycosidic linkages is currently unknown. GTFO of the probiotic bacterium *L. reuteri* ATCC 55730 represents another reuteransucrase and was found to produce a higher proportion of (α1 → 4) linkages (80%) and less (α1 → 6) linkages (20%) in its polysaccharide^38^. GTFO shows high similarity (80%) at the amino acid level with GTFA^38^. However, it does not produce alternating (α1 → 4) and (α1 → 6) linkages. In order to study the determinants for the different linkage specificity of GTFA and GTFO, and to elucidate the mechanism of alternating linkage synthesis, we performed a site-directed mutagenesis study with GTFO, targeting the amino acid residues that are probably involved in linkage specificity determination and are different between GTFO and GTFA. Specifically, we mutated GTFO amino acids to the corresponding residues present in GTFA. Our results show that loop977 (^970^DGKGYKGA^977^) and helix α4 (^1083^VSLKGA^1088^) residues are the major determinants for the linkage specificity difference between GTFO and GTFA. Changing these residues of GTFO to the corresponding residues of GTFA enabled it to synthesize a large amount of alternating (α1 → 4) and (α1 → 6) linkages. Furthermore, we show that A977 in loop977 and V1083 and G1087 in helix α4 are the most critical residues.

## Results and Discussion

### Construction and production of GTFO-∆N mutants

**Figure 1:**
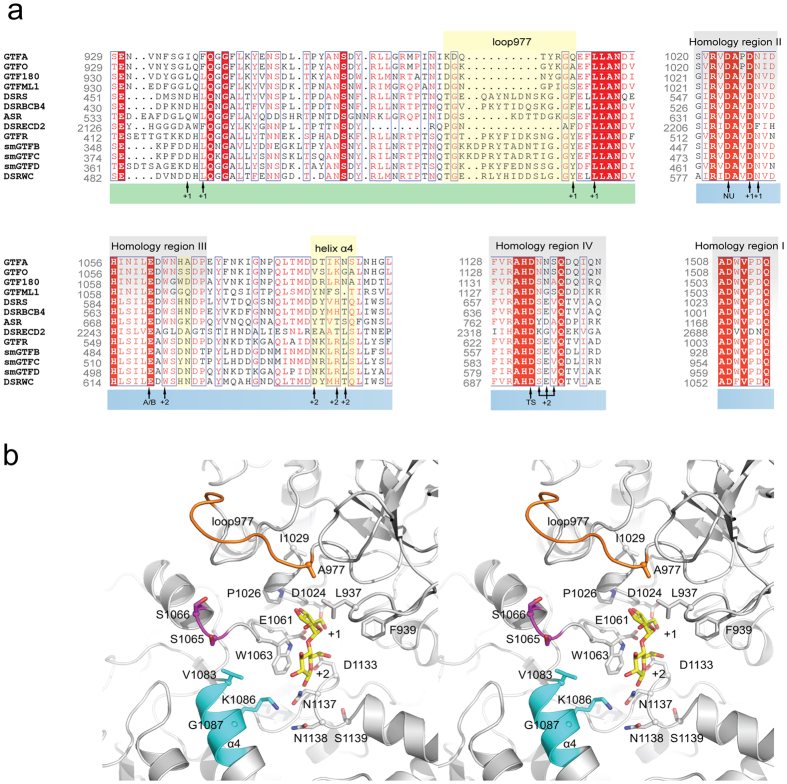
(**a**) Partial alignment of glucansucrase amino acid sequences of family GH70. Residues from domain A are highlighted with blue at the bottom and residues from domain B are highlighted in green at the bottom. Homology regions I to IV of glucansucrases are highlighted in gray. Regions targeted for mutagenesis in this study are highlighted in yellow. Catalytic residues and residues involved in shaping acceptor binding sites +1 and +2 are indicated. (**b**) Stereo figure showing a model of the GTFO active site in cartoon representation, with the regions and residues targeted for mutation highlighted in color: loop977 (residues ^970^DGKGYKGA^977^, orange), S1065-S1066 (magenta) and the N-terminal part of helix α4 (residues ^1083^VSLKGA^1088^, cyan). The maltose bound in subsites +1 and +2 of *L. reuteri* 180 GTF180-ΔN^16^ is shown with yellow carbon atoms. The catalytic residues of GTFO (D1024, E1061 and D1133), and other residues surrounding the active site, are shown in stick representation.

Residues in homology regions I-IV in domain A are critical for linkage specificity determination of glucansucrase enzymes, as shown in previous mutagenesis studies^15^,^39^,^40^,^41^,^42^,^43^. Moreover, residues from domain B have been shown to shape the acceptor binding sites and hence determine the linkage specificity^10^,^16^,^41^,^44^,^45^. The amino acid sequences of these regions in GTFO and GTFA were compared by alignment with the other glucansucrase enzymes from LAB (Fig. 1a). The amino acid residues in these regions that are different between GTFO and GTFA were selected for mutagenesis. In homology region III, following the acid/base catalyst, GTFO differs in two amino acid residues (S1065:S1066, SS) with GTFA (H1065:A1066, HA)). Residues S1065:S1066 of GTFO-∆N were mutated to H and A, respectively, to mimic those of GTFA. At acceptor substrate binding subsite +1 (Fig. 1), GTFO and GTFA contain A977 and Q977, respectively; the corresponding residues in GTF180 (A978 in domain B) has been shown to be important for linkage specificity^45^. The loop containing this residue (^970^DGKGYKGA^977^, loop977, Fig. 1a, highlighted in yellow) differs between glucansucrases. In view of the possible synergistic effects, loop977 in GTFO was mutated to the corresponding residues of GTFA to evaluate its role in determining linkage specificity. In addition, residue A977 was mutated separately to Q to investigate its effect on linkage specificity. Another residue at subsite +1, L937 of GTFO, differs from the corresponding residue in GTFA (I938), but was not mutated in the present study since Leu and Ile are highly similar and likely play similar roles in linkage specificity determination. At subsite +2, the glucosyl moiety of maltose has interactions with residue W1063 in homology region III, N1134:N1135:S1136 in homology region IV following transition state stabilizer D1136, and V1083:K1086:G1087 from helix α4 following the homology region III (Fig. 1)^16^. Residue W1063 as well as residues following the transition state stabilizer D1136 in homology region IV are identical in GTFO and GTFA. However, helix α4 varies between GTFO and GTFA. In particular, two of the three residues (V1083 and G1087) from helix α4, which participate in shaping the acceptor binding site +2 (Fig. 1b), are different. We mutated residues in a segment of helix α4 (residues ^1083^VSLKGA^1088^, Fig. 1a, highlighted in yellow) of GTFO to those present in GTFA. Residue V1083 and G1087 were individually or jointly mutated in combination with the other mutations. In addition, these three groups of residues (loop977, SS, α4) were targeted combinatorically, anticipating an interplay of different residues in linkage specificity determination. Apart from the well-conserved homology regions (I to IV), three additional regions (V to VII) have been proposed to be the determinants for reaction specificity of GH13 family enzymes^46^. In a previous study regarding the starch acting enzymes GTFB and GTFC (GH70 4,6-α-glucanotransferases), GH70 regions V-VII were compared with those of GH13 family α-amylases^47^. Homology regions V, VI and VII of GTFA and GTFO are almost identical, only differ in one residues of homology region V (Val985 in GTFO and Ile986 in GTFA). Homology regions V-VII therefore are highly unlikely to be involved in the linkage specificity difference between GTFA and GTFO. We thus have chosen not to include homology regions V-VII in the present study. The primers for site-directed mutagenesis are listed in Table 1 and the list of mutants constructed in this study is shown in Table 2. All GTFO-∆N mutant proteins showed comparable expression levels compared to that of wild-type GTFO-∆N.

### Activity of GTFO-∆N mutants, wild-type GTFO-∆N and GTFA-∆N enzymes

As shown in Table 2, compared to GTFO-∆N, GTFA-∆N showed higher activities (about 2 fold). GTFO mutant enzymes with an exchanged loop977 (lp977) retained relatively low activities (10% or less). However, mutation A977Q alone had no negative effects on activity, suggesting that exchanging multiple residues in loop977 caused more severe rearrangements in the active site, thereby reducing the activity. Mutant GTFO-∆N enzymes A977Q/V1083D, A977Q/G1087N and A977Q/V1083D/G1087N showed increased activity, to values near that of GTFA-∆N. The other mutant enzymes displayed activity values similar to that of wild-type GTFO-∆N at 100 mM sucrose. GTFO has been characterized as a highly hydrolytic reuteransucrase in our previous studies^38^. Under the conditions used in this study, GTFO-∆N hydrolyzed 43% of sucrose into glucose and fructose while this value is only 9% for GTFA-∆N (Table 2). The hydrolysis of all mutant GTFO-∆N enzymes was only slightly reduced and remained relatively high compared to that of GTFA-∆N (Table 2). Thus, the main determinants for the high hydrolytic activity of GTFO are not the residues targeted in this study. Other regions different between GTFA and GTFO must be responsible, and it is worth to investigate those regions for their role in determining the high hydrolysis/transglycosylation ratio of GTFO.

### Effects of mutations on linkage specificity using sucrose as substrate

All the mutant enzymes constructed in this study retained the ability to synthesize polysaccharide from sucrose. The linkage composition of polysaccharides produced was determined by ^1^H NMR and methylation analysis (Table 2). ^1^H NMR analysis showed that wild-type GTFO-∆N produced polysaccharides consisting of 79% of (α1 → 4) linkages and 21% of (α1 → 6) linkages, while the polysaccharides synthesized by GTFA-∆N contain 58% (α1 → 4) linkages and 42% of (α1 → 6) linkages (Table 2, Fig. 3a), in agreement with our previous studies^34^,^38^. Mutant GTFO-∆N lp977 and GTFO-∆N α4 both produced polysaccharides with an elevated level of (α1 → 6) linkages (33% and 36%, respectively). However, GTFO-∆N mutant S1065H:S1066A synthesized polysaccharides similar to those produced by wild-type GTFO-∆N. These results demonstrate that loop977 and helix α4 play an important role in determining the linkage specificity of GTFO and GTFA, but not residues S1065 and S1066. Mutant GTFO-∆N A977Q synthesized polysaccharides containing almost the same amount of (α1 → 6) linkages as that of mutant GTFO-∆N lp977, indicating that residue A977 is the major determinant for linkage specificity in loop977. This is also supported by the observation that A977 of GTFO is located close to the acceptor substrate binding site +1 (Fig. 1b)^48^.

Although mutations of lp977 and α4 residues resulted in the production of polysaccharides with a higher percentage of (α1 → 6) linkages (about 30%), these values remained low compared to that of GTFA-∆N (42%). We also combined these three mutated regions and investigated their effects on linkage specificity (Table 2). Mutant GTFO-∆N lp977/SS-HA/α4 (combining all three mutated regions) catalyzed the synthesis of polysaccharides with 40% (α1 → 6) linkages and 60% (α1 → 4) linkages, which is almost the same as observed for GTFA-∆N. Apparently, the synergetic effects of the mutations at the three targeted regions caused a further increase of (α1 → 6) linkages in the polysaccharides from 36% to 40%. The ^1^H NMR spectrum of the polysaccharide produced by GTFO-∆N lp977/SS-HA/α4 is highly similar to that of GTFA-∆N at both the anomeric region and the bulk region (Fig. 3a). Mutant GTFO-∆N A977Q/SS-HA/α4 produced a polysaccharide identical to that of mutant GTFO-∆N lp977/SS-HA/α4, confirming that A977 is the crucial determinant in the loop977. Mutant GTFO-∆N lp977/α4 and GTFO-∆N A977Q/α4 produced identical polysaccharides as GTFO-∆N lp977/SS-HA/α4 and GTFO-∆N A977Q/SS-HA/α4, respectively. These results confirmed again that residues S1063:S1064 are not involved in the determination of linkage specificity and in the observed synergistic effects of the three mutated regions. This is further supported by the observation that mutant GTFO-∆N lp977/SS-HA and GTFO-∆N SS-HA/α4 produced polysaccharides with the same amount of (α1 → 4) and (α1 → 6) linkages as GTFO-∆N lp977 and GTFO-∆N α4, respectively. To further investigate the major determinants in helix α4, residues V1083 and G1087 were mutated individually or jointly in combination with mutation A977Q in view of their close distance to acceptor binding site +2 (Fig. 1b)^16^. Mutants GTFO-∆N A977Q/V1083D and GTFO-∆N A977Q/G1087N synthesized 36% and 34% of (α1 → 6) linkages in their polysaccharides, respectively. Mutant GTFO-∆N A977Q/V1083D/G1087N produced a higher amount of (α1 → 6) linkages (38%, almost the same as that of GTFO-∆N A977Q/α4) in its polysaccharide compared to that of GTFO-∆N A977Q/V1083D and GTFO-∆N A977Q/G1087N. These results indicate that residues V1083 and G1087 in helix α4 are the crucial residues for determining the different linkage specificity of GTFO and GTFA. Particularly, the synergistic effect of residues V1083 and G1087 was shown to be important. This may be explained by the fact that V1083:K1086:G1087 correspond to residues in GTF180 that interact with the C2 hydroxyl group of the +2 glucosyl unit of maltose via indirect hydrogen bonds through the same water molecule^16^. Similar interactions may occur in GTFO and GTFA, explaining the observed effects on linkage specificity. To conclude, residues A977, V1083 and G1087 of GTFO are the main determinants for the different linkage specificity of GTFO and GTFA.

The amounts of (α1 → 6) linkages in the polysaccharides produced by mutants GTFO-∆N lp977/SS-HA/α4, GTFO-∆N A977Q/SS-HA/α4, GTFO-∆N lp977/α4, GTFO-∆N A977Q/α4 and GTFO-∆N A977Q/V1083D/G1087N were slightly lower compared to that of GTFA-∆N. Methylation analysis was performed with polysaccharides produced by all the mutants constructed in this study (Table 2). The results showed that the polysaccharide produced by wild-type GTFO-∆N contained a large amount of → 4)Glc*p*(1 → units (69%), and a low amount of → 6)Glc*p*(1 → units (9%) and → 4,6)Glc*p*(1 → units (11%). The polysaccharides produced by GTFA-∆N are composed of 45% → 4)Glc*p*(1 → unit, 27% → 6)Glc*p*(1 → units and 15% → 4,6)Glc*p*(1 → units. Compared to GTFA-∆N, the GTFO-∆N mutants mentioned above produced less amounts of → 6)Glc*p*(1 → units (approximate 22%) and slightly larger amounts of → 4,6)Glc*p*(1 → units (approximately 17%) (Table 2).

### Pullulanase digest of polysaccharides

**Figure 2:**
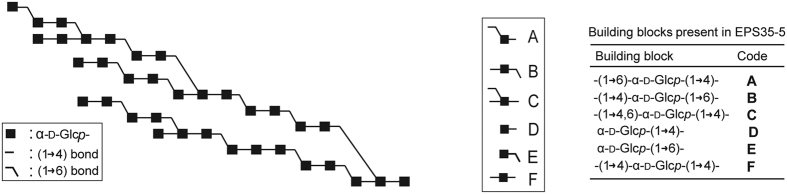
Composite structure of the reuteran polysaccharide produced by the GTFA enzyme of *Lactobacillus reuteri* 121^34^.

**Figure 3:**
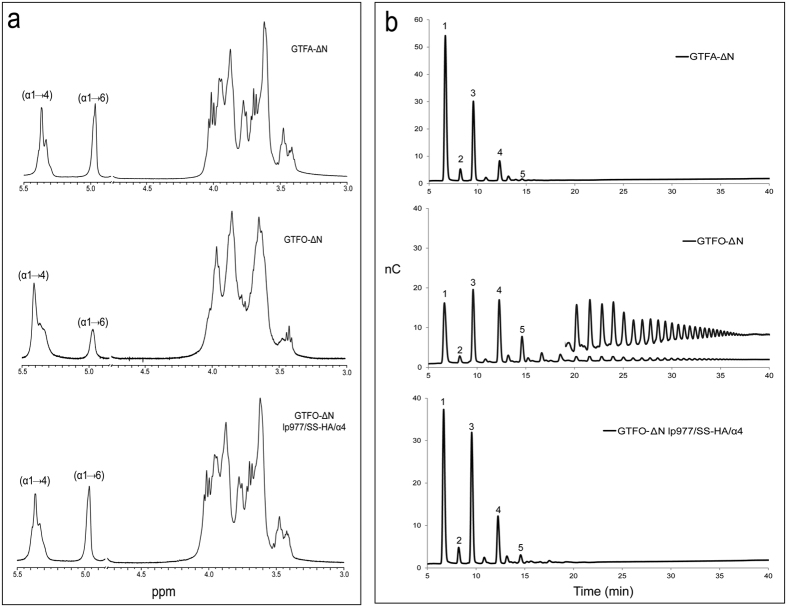
(**a**) ^1^H NMR spectra of the polysaccharides produced by the wild-type GTFA-ΔN, GTFO-ΔN and mutant GTFO-ΔN lp977/SS-HA/α4. (**b**) HPAEC-PAD (Carbo-Pac PA-1) analysis of the oligosaccharides produced by digesting the polysaccharides synthesized from sucrose by wild-type GTFA-ΔN, GTFO-ΔN and mutant GTFO-ΔN lp977/SS-HA/α4 using pullulanase M1 of *Klebsiella planticola*. 1: maltose; 2: panose; 3: maltotriose; 4: maltotetraose; 5; maltopentaose.

In a previous study we showed that the polysaccharide produced by GTFA-∆N is susceptible to hydrolysis by pullulanase M1 of *Klebsiella planticola*, which specifically hydrolyzes the (α1 → 6) linkage in the sequence of (-)α-D-Glc*p*-(1 → 4)-α-D-Glc*p*-(1 → 6)-34. Pullulanase M1 digestion of the polysaccharide produced by GTFA-∆N resulted in the production of maltose, panose, maltotriose, maltotetraose and maltopentaose (Fig. 3b). The significant production of maltose indicates that mostly alternating (α1 → 4) and (α1 → 6) linkages are present in the GTFA-∆N polysaccharide (Fig. 2), as reported in our previous study^34^. In contrast, pullulanase M1 digestion of the polysaccharide produced by GTFO-∆N mainly produced a range of malto-oligosaccharides from DP2 up to DP40 as detected by HPAEC-PAD (Fig. 3b) and MALDI-TOF-MS. This suggests that mostly continuous (α1 → 4) linkages are present in the GTFO-∆N polysaccharide. Pullulanase M1 digestion of the polysaccharides produced by all GTFO-∆N mutant enzymes (except mutant GTFO-∆N SS-HA) resulted in similar product profiles to that of GTFA-∆N, with an increase of short chain oligosaccharides and absence of malto-oligosaccharides DP > 6 (Fig. 3b), compared to wild-type GTFO-ΔN. Interestingly, the highly increased production of maltose suggests that the GTFO-∆N mutant enzymes synthesize mostly alternating (α1 → 4) and (α1 → 6) linkages, like GTFA-∆N. Pullulanase M1 digestion of the polysaccharides formed by GTFA-∆N yielded 45.9% maltose (based on glucose unit) while this value was only 8.7% for GTFO-∆N (Table 2). All GTFO-ΔN mutant enzymes (except mutant GTFO-∆N SS-HA) incorporated higher amounts of glucosyl units (from 25 to 36%) in the form of maltose, indicating a large increase of alternating (α1 → 4) and (α1 → 6) linkage synthesis. The highest maltose recoveries based on glucosyl units (Table 2) were observed for the polysaccharides produced by GTFO-∆N lp977/SS-HA/α4, GTFO-∆N A977Q/SS-HA/α4, GTFO-∆N lp977/α4, GTFO-∆N A977Q/α4 and GTFO-∆N A977Q/V1083D/G1087N, which also produced polysaccharides with similar ^1^H NMR spectra to that of GTFA-∆N. Thus, we conclude that loop977 and helix α4 are critical for determining the alternating (α1 → 4) and (α1 → 6) linkage synthesis of GTFA. Within the loop977 and helix α4, residues A977, V1083 and G1087 are the major determinants for the different linkage specificity of the GTFO and GTFA polysaccharide products.

### Oligosaccharide synthesis from maltose and sucrose

In the presence of sucrose (donor substrate) and maltose (acceptor substrate), GTFO-∆N and GTFA-∆N mainly catalyze the synthesis of panose, maltotriose, glucosyl-(α1 → 4)-panose, glucosyl-(α1 → 6)-(α1 → 4)-panose and maltotetraose, by elongation of maltose at its non-reducing end with a glucosyl moiety from sucrose, introducing (α1 → 6) or (α1 → 4) linkages (Table 3)^15^,^37^,^38^. Compared to GTFA-∆N, GTFO-∆N synthesized less panose and more maltotriose (Table 3), which can be explained by their different linkage specificities. Compared to wild-type GTFO-∆N, mutant enzymes containing mutations in loop977 were not affected in the amounts of glucosyl-(α1 → 4)-panose (13%) and glucosyl-(α1 → 6)-(α1 → 4)-panose (1%) produced; in contrast, they showed an increase in panose production (to about 45%), and a reduction in maltotriose production (to about 7%). Apparently, these mutations in loop977 impaired the ability of the enzymes to synthesize successive (α1 → 4) linkages. The results also show that these mutant enzymes are capable of forming (α1 → 4) linkages after (α1 → 6) linkages (apparent from the high amount of glucosyl-(α1 → 4)-panose produced), but that these mutations in general negatively affect the ability to synthesize (α1 → 6) linkages after (α1 → 4) linkages (apparent from the low amounts of glucosyl-(α1 → 6)-(α1 → 4)-panose). Mutant enzymes only involving helix α4 residues produced similar amounts of panose and maltotriose, slightly lower amount of glucosyl-(α1 → 4)-panose and higher amounts of glucosyl-(α1 → 6)-(α1 → 4)-panose, suggesting that these mutations enhanced the ability of enzymes to synthesize alternating (α1 → 6) and (α1 → 4) linkages, however, still retained the relative high ability to form successive (α1 → 4) linkages. Mutant enzymes containing mutations both in loop977 and helix α4 showed both reduced amounts of maltotriose production and increased amounts of glucosyl-(α1 → 6)-(α1 → 4)-panose production, suggesting a strongly enhanced ability to form alternating (α1 → 6) and (α1 → 4) linkages. These results demonstrate that loop977 and helix α4 play different roles in the observed linkage specificity, and that the combination of mutations in both regions is required to enable GTFO to synthesize alternating (α1 → 6) and (α1 → 4) linkages. Mutations in residues V1083 and G1087 in GTFO-∆N helix α4, which already have been shown to be involved in polysaccharide linkage specificity, also seem to affect the product profiles from sucrose and maltose. Furthermore, V1083D is the major mutation that determines the synthesis of alternating (α1 → 4) and (α1 → 6) linkages as shown by the production of higher amounts of glucosyl-(α1 → 6)-(α1 → 4)-panose. In contrast, GTFO-∆N mutant enzymes involving only S1063:S1064 produced equal amounts of panose, maltotriose, glucosyl-(α1 → 4)-panose, glucosyl-(α1 → 6)-(α1 → 4)-panose and maltotetraose as wild-type GTFO-∆N (Table 3). Apparently, residues S1063:S1064 are not involved in determining linkage specificity.

### Oligosaccharide synthesis from isomaltose and sucrose

With the availability of sucrose (donor substrate) and isomaltose (acceptor substrate), GTFO-∆N and GTFA-∆N catalyze the synthesis of isopanose as major product, and minor amounts of isomaltotriose, from the elongation of isomaltose at the non-reducing end with (α1 → 4) linkages and (α1 → 6) linkages, respectively (Table 4)^15^,^38^. Isopanose is capable of acting as acceptor to be elongated further at its non-reducing end with (α1 → 6) linkages, resulting in the production of glucosyl-(α1 → 6)-isopanose [α-D-Glc*p*-(1 → 6)-α-D-Glc*p*-(1 → 4)-α-D-Glc*p*-(1 → 6)] with alternating (α1 → 6) and (α1 → 4) linkages. GTFA-∆N produced 16.5% of isopanose and 12.3% of glucosyl-(α1 → 6)-isopanose in the presence of 100 mM sucrose and 100 mM isomaltose. Compared to GTFA-∆N, GTFO-∆N produced a larger amount of isopanose (35.5%) and only a tiny amount of glucosyl-(α1 → 6)-isopanose (1.9%). These results are in agreement with the previous observation that GTFA-∆N has a higher capability to form alternating (α1 → 6) and (α1 → 4) linkages than GTFO-∆N^38^. Mutations in GTFO-∆N helix α4 (except for GTFO-∆N A977Q/G1087N) increased the ability to synthesize alternating (α1 → 6) and (α1 → 4) linkages, shown by the increased levels of glucosyl-(α1 → 6)-isopanose produced. On the other hand, mutations only targeting loop977 or S1063:S1064 showed less or no increase in the production of glucosyl-(α1 → 6)-isopanose. In accordance with earlier conclusions, residues S1063:S1064 are not involved in the linkage specificity hence also do not affect the product profiles from sucrose and isomaltose. Mutations in loop977 have less effect on the product spectrum from sucrose and isomaltose, in agreement with the earlier analysis of produced polysaccharides (Tables 2 and 4).

### Oligosaccharide synthesis from sucrose and panose or maltotriose

**Figure 4:**
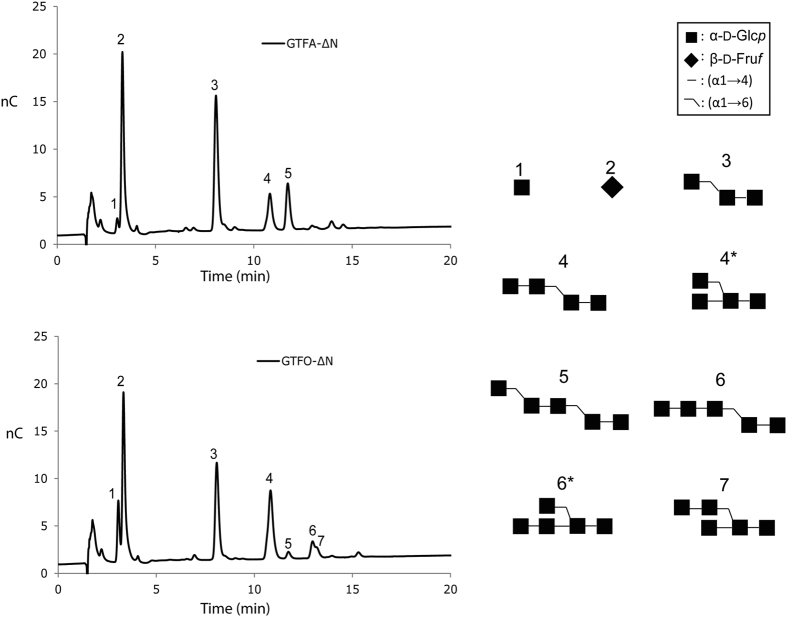
HPAEC-PAD profiles of the oligosaccharide mixtures generated from 100 mM sucrose and 100 mM panose with the GTFA-∆N and GTFO-∆N enzymes. Oligosaccharide structures identified are included.

Sucrose (donor substrate, 100 mM) and panose (acceptor substrate, 100 mM) were incubated with GTFA-∆N or GTFO-∆N in order to further explore their product specificity. The major oligosaccharides produced were isolated and characterized by MALDI-TOF-MS and 1D/2D ^1^H/^13^C NMR spectroscopy. Structures of formed oligosaccharides are presented in Fig. 4. Oligosaccharides 4, 4* and 5 show 1D ^1^H spectra identical to the oligosaccharides characterized in our previous studies^37^ (Supplementary Fig. S1). Identification of oligosaccharides 6, 6* and 7 was based on the previously developed structural-reporter-group concept for α-glucans^34^,^37^,^49^. As an example of the interpretation of the various ^1^H and ^13^C NMR assignments, the 1D ^1^H and 2D (TOCSY, ROESY and HSQC) NMR spectra of oligosaccharide 7 (*m/z,* 829.5 [M + H]^+^, according to MALDI-TOF-MS), are presented in Supplementary Fig. S2. For further NMR data see Supplementary Table S1.

Incubation of sucrose (donor substrate, 100 mM) and panose (acceptor substrate, 100 mM) with GTFA-∆N yielded glucosyl-(α1 → 4)-panose and glucosyl-(α1 → 6)-(α1 → 4)-panose as the major oligosaccharide products, revealing its capability of synthesizing alternating (α1 → 4) and (α1 → 6) linkages. Minor amounts of a branched oligosaccharide [4*, α-D-Glc*p*-(1 → 4)-[α-D-Glc*p*-(1 → 6)]-α-D-Glc*p*-(1 → 4)-α-D-Glc*p*-(1 → 4)] were also synthesized by GTFA-∆N. The large amount of glucosyl-(α1 → 4)-panose and the low amount of glucosyl-(α1 → 6)-(α1 → 4)-panose produced by GTFO-∆N under the same conditions indicates that this enzyme prefers to form (α1 → 4) linkages over alternating (α1 → 4) and (α1 → 6) linkages, similar to when maltose or isomaltose were used as acceptors. The production of compound 6, 6* and 7 shows that GTFO-∆N continues to elongate glucosyl-(α1 → 4)-panose with (α1 → 4) linked glucose units.

**Figure 5:**
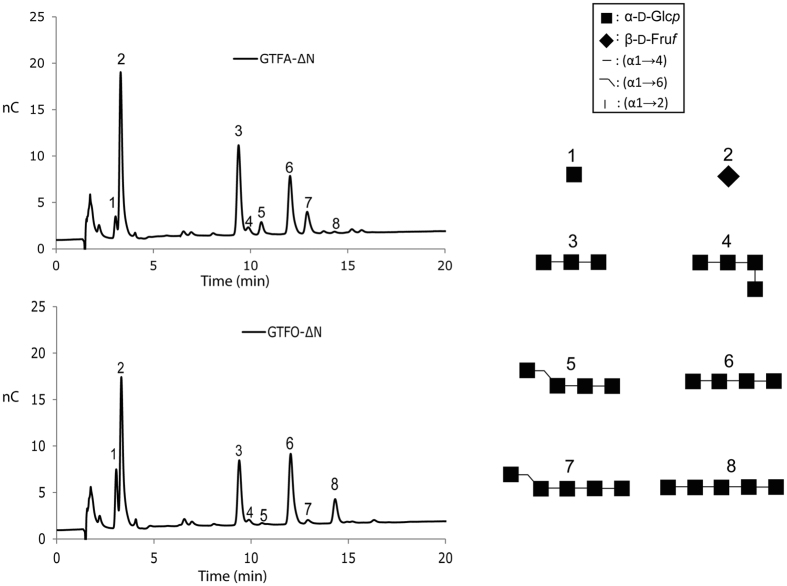
HPAEC-PAD profiles of the oligosaccharide mixture generated from 100 mM sucrose and 100 mM maltotriose with the GTFA-∆N and GTFO-∆N enzymes. Oligosaccharide structures identified are included.

Isolation and characterization of the major oligosaccharides produced from the incubation of GTFA-∆N or GTFO-∆N with sucrose (donor substrate, 100 mM) and maltotriose (acceptor substrate, 100 mM) showed that maltotetraose is the major product in case of GTFA-ΔN (Fig. 5 and Supplementary Fig. S3). With an (α1 → 4) linkage present between acceptor binding subsites +1 and +2, GTFA-∆N prefers to form an (α1 → 6) linkage as observed from the reaction using maltose as acceptor (Table 3). This observation with maltotriose indicated that the linkage between the +2 and +3 subsites, and the amino acid residues of the +3 subsite (or even further sites) are also determinants of linkage specificity. Two (α1 → 6) linkage-elongated oligosaccharides (compounds 5 and 7, Fig. 5) were also formed, but in clearly lower amounts. In case of GTFO-∆N and maltotriose, mainly (α1 → 4) linkages were formed, resulting in synthesis of maltotetraose and maltopentaose. Only minor amounts of compounds 5 and 7 ((α1 → 6) linkage-elongated oligosaccharides) were produced, reflecting the preference of GTFO-∆N for formation of (α1 → 4) linkages.

### Comparison of GTFO and GTFA amino acid sequence and their products sheds light on alternating linkage formation

GTFA of *L. reuteri* 121 has been shown to produce a large amount of alternating (α1 → 4) and (α1 → 6) linkages^34^,^37^. GTFO of the probiotic bacterium *L. reuteri* ATCC55730, shows a high sequence similarity (80%) with GTFA of *L. reuteri* 121 and also synthesizes (α1 → 4) and (α1 → 6) linkages in the α-glucans but at a clearly different ratio compared to GTFA^38^. A comparison of GTFA and GTFO, therefore, provides an excellent opportunity to identify the amino acid residues that are responsible for the difference between their alternating and non-alternating (α1 → 4) and (α1 → 6) linkage formation, respectively. Since the orientation of the acceptor substrate prior to transglycosylation determines the linkage to be made, residues forming the acceptor binding sites must determine the linkage specificity in glucansucrase enzymes. Earlier studies have identified a number of residues that contribute to linkage specificity. For example, GH70 glucansucrase enzymes possess four conserved regions (I-IV), also found in related GH13 family enzymes (Fig. 1a)^8^,^9^,^10^,^14^,^15^. Amino acid residues following the catalytic nucleophile D1024 (GTFO numbering) in homology region II are conserved (1025A-V-D-N-V1029) in most glucansucrases whereas GTFA and GTFO have P1026 and I1029 at their corresponding position^15^. In homology region IV, following the transition state stabilizing residue, most glucansucrases from *Leuconostoc* and *Streptococcus* have the S-E-V tripeptide; GTFO and GTFA, however, have a different tripeptide (N-N-S)^15^. Mutation of this tripeptide in GTFA to the conserved S-E-V motif resulted in a drastic increase of (α1 → 6) linkages in the polysaccharides synthesized^15^; combination of N1134S:N1135E:S1136V with P1029V:I1029V caused an even further increase in the amount of (α1 → 6) linkages^15^, suggesting that these residues are important for (α1 → 4) linkage specificity of GTFA. Since GTFO and GTFA have the same N-N-S motif, these residues likely do not play a role in determining their differences in linkage specificity. In homology region III, GTFO and GTFA differ in the two residues at positions 1065 and 1066, containing 1065S:1066S and 1065H:1066A, respectively. Although mutating these two residues of GTFA to those of GTFO showed no effect on linkage specificity^15^, we applied the reverse change in this study in view of the potential synergistic effects of amino acid residues around the acceptor binding site. However, also in this case our results showed that residues 1065S:1066S of GTFO are not involved in linkage specificity determination (Table 2).

In addition to residues from the conserved regions I-IV, the crystal structure of glucansucrase GTF180-∆N in complex with the acceptor substrate revealed that other regions, especially residues from domain B, also shape the acceptor binding site and hence may contribute to linkage specificity^16^. For example, at subsite +1, the non-reducing end glucosyl unit of maltose is surrounded by residues from domain A (D1028 and N1029 in homology region II, GTF180 numbering) and residues from domain B (L938, L940, A978 and L981)^16^. Mutagenesis studies showed that these residues are indeed involved in determining linkage specificity and enzyme activity in GTF180^44^,^45^. In GTFO and GTFA the residues corresponding to A978 are A977 and Q977, respectively. Residue A977 is located in a loop (loop977, residues ^970^DGKGYKGA^977^, Fig. 1a) in domain B. Given the fact that this loop varies in length and composition between different glucansucrases, one may expect the same residues to affect their linkage specificity. Indeed, mutating loop977 of GTFO to the corresponding residues of GTFA enabled the GTFO mutants to synthesize alternating (α1 → 4) and (α1 → 6) linkages (Table 2), confirming the importance of this loop. Other residues at subsite +1 from domain A and B are identical or similar in GTFO and GTFA (Fig. 1a) and therefore likely do not account for their linkage specificity difference. Interestingly, GTFO and GTFA both contain a phenylalanine at position 939 (Fig. 1a), while all other glucansucrase enzymes (except DSRECD2) contain a leucine; for example, in GTF180, L940 was shown to be critical for linkage specificity determination^44^. We speculate that, in view of their identity in GTFO and GTFA, this F939 residue accounts for the (α1 → 4) linkage synthesis in GTFO and GTFA. At subsite +2, residues following the transition state stabilizer (the N-N-S motif) have been shown to be important in linkage specificity in previous mutagenesis studies^15^,^39^,^40^,^41^,^42^,^43^, and are located at the O1/O5/O6 side of the reducing end glucosyl moiety of maltose (Fig. 1b), explaining their effects on linkage specificity^16^. At the other (O2/O3) side of the maltose, residues D1085, R1088 and N1089 of GTF180 all form water-mediated hydrogen bonds to the C2 hydroxyl group of the +2 glucosyl moiety^16^; also these residues have been shown to be involved in linkage specificity determination in GTF180 and DSRS^50^,^51^. Our sequence alignment analysis showed that the corresponding residues vary greatly between different glucansucrase enzymes. To study their importance in GTFO, these residues and their adjacent residues were mutated to those present in GTFA; we found that this region (helix α4, residues ^1083^VSLKGA^1088^, Fig. 1b) contributes to the linkage specificity difference between GTFA and GTFO; residues V1083 and G1087 are most critical in this regard. Our detailed characterization shows that the mutations alter the product linkage type and distribution of GTFO, and thus clearly shows that that loop977 near subsite +1 and helix α4 near subsite +2 (Fig. 1) are the main determinants of the linkage specificity differences between GTFO and GTFA. Mutating these two regions in GTFO to the corresponding regions of GTFA enable enzymes capable of synthesizing alternating (α1 → 4) and (α1 → 6) linkages. Likely, these regions determine the orientation of incoming acceptor substrates. For example, one may speculate that the introduction of residues Q977 (in loop977) and N1087 (in helix α4), replacing residues A978 and G1085, respectively, adds hydrogen-bonding capabilities in the acceptor binding space. This may confine the orientation of acceptor substrates in such a way that only alternating linkage types are formed (depending on the previously formed linkage type).

Analysis of the oligosaccharides synthesized by the mutant enzymes using maltose and isomaltose as acceptor substrates showed that loop977 and helix α4 synergistically contribute to the alternating (α1 → 4) and (α1 → 6) linkage synthesis. Mutations in loop977 in GTFO to corresponding GTFA residues favor alternating linkage synthesis by impairing successive (α1 → 4) linkages synthesis. In contrast, mutations of helix α4 in GTFO to corresponding GTFA residues enable alternating linkage synthesis by favoring the formation of (α1 → 6) linkages. Moreover, using panose as acceptor substrate, GTFA catalyzes the synthesis of oligosaccharides with alternating (α1 → 4) and (α1 → 6) linkages. When maltotriose is used as acceptor substrate, it is elongated with (α1 → 4) linkages by GTFA, yielding maltotetraose as major product. These results indicate that also the +3 subsite contributes to determining linkage specificity. Currently, no crystal structures of GTFA are available with longer acceptor substrates (DP > 3) to support these observations, and molecular docking studies gave no clear results due to the low resolution of the crystal structure^48^. Another glucansucrase capable of forming alternating linkages is the (α1 → 3)/(α1 → 6) specific ASR. Comparing the amino acid residues described above in GTFA and ASR showed that they are different, indicating that they may use a different mechanism to form alternating linkages. For example, the tripeptide (Y-D-A) following the transition state stabilizer in ASR was shown to be important for alternating (α1 → 3)/(α1 → 6) linkages synthesis; the tyrosine residue in this tripeptide was proposed to act as a second stacking platform at subsite +2^42^. In GTFA the absence of such an aromatic residue (the corresponding tripeptide is N-N-S) may account for its different linkage type preference [(α^1^ → 4)/(α1 → 6)].

In conclusion, we showed that loop977 (residues ^970^DGKGYKGA^977^, Fig. 1) and helix α4 (residues ^1083^VSLKGA^1088^, Fig. 1) are the main determinants for the linkage specificity differences between GTFO and GTFA, in particular regarding the alternating (α1 → 4)/(α1 → 6) linkages synthesis in GTFA. In addition, more remote sites (i.e. +3) are involved in the determination of alternating linkage synthesis as shown by the oligosaccharide synthesis studied using panose and maltotriose as acceptor substrates. Combining our results with those of previous mutagenesis studies provides evidence that the amino acid residues at acceptor substrate binding sites (+1, +2, +3…) together form a distinct physicochemical micro-environment that determines the alternating or non-alternating linkage synthesis in the (α1 → 4)/(α1 → 6)-specific glucansucrases GTFA and GTFO. Crystallography studies of GTFA in complex with longer acceptor substrate are in progress and will shed further light on the mechanism of alternating linkage synthesis.

## Methods

### Sequence alignments

ClustalW2 was used to align the amino acid sequences of glucansucrases from *Lactobacillus*: GTFA (Q5SBL9) of *L. reuteri* 121, GTFO (Q4JLC7) of *L. reuteri* ATCC 55730, GTF180 (Q5SBN3) of *L. reuteri* 180, GTFML1 (Q5SBN0) of *L. reuteri* ML1; glucansucrases from *Leuconostoc*: DSRS (Q9ZAR4) of *L. mesenteroides* NRRL B-512F, DSRBCB4 (D2CFL0) of *L. mesenteroides* B-1299CB4, alternansucrase ASR (Q9RE05) from *L. mesenteroides* NRRL B-1355, DSRE CD2 (Q8G9Q2) of *L. mesenteroides* NRRL B-1299; glucansucrase from *Streptococcus*: GTFR (Q9LCH3) of *S. oralis* ATCC10557, smGTFB (P08987) of *S. mutans* GS 5, smGTFC (P13470) from *S. mutans* GS 5, smGTFD (P49331) of *S. mutans* GS 5; and glucansucrases from *Weissella*: DSRWC (B9UNL6) of *Weissella cibaria* CMU. The aligned sequences were then used as input for ESPript (http://espript.ibcp.fr) for alignment with the GTFA-ΔN crystal structure (PDB: 4AMC)^48^,^52^.

### Modeling

A homology model of GTFO was constructed using residues 747–1781 of GTFO (predicted to contain domains A, B, C, IV and V), and the crystal structure of GTFA-ΔN^53^ as a template for the one-to-one threading protocol of Phyre (http://www.sbg.bio.ic.ac.uk/phyre2/)^54^; sequence identity between the template and the model is 68%. The resulting GTFO model has a confidence of 100% and covers residues 749–1763. Acceptor substrate binding subsites +1 and +2 were mapped by superimposition with the crystal structure of the GTF180-ΔN - maltose complex (PDB 3KLL)^16^.

### Site-directed mutagenesis

The site-directed mutations were introduced using a site-direct mutagenesis kit (Stratagene, La Jolla, USA). Truncation of the N-terminal variable domain (GTFO-ΔN, encoding amino acids 744–1781) had no significant effect on the activity and product spectrum of GTFO^38^. Therefore, plasmid pET15b-GTFO-ΔN, constructed in a previous study^38^, was used as template for site-directed mutagenesis. The primers used for site-directed mutagenesis are summarized in Table 1. Mutations of loop977 (^970^DGKGYKGA^977^) were performed in two steps: (1) Residues ^974^YKGA^977^ were mutated in the first step; (2) Residues ^970^DGKG^973^ were mutated in the second step using the mutated plasmid obtained in the first step as template. The other mutants were obtained by combining mutations using appropriate primers and templates. All the mutations were verified by nucleotide sequencing (GATC, Konstanz, Germany).

### Enzyme expression and purification

Overnight cultures of *E. coli* BL21 DE3 star strains containing the appropriate expression plasmids with *gtfA*-ΔN, *gtf*O-ΔN and mutant *gtf*O-ΔN genes were inoculated in fresh LB media with 100 μg/ml Ampicillin. Cultures were grown at 37 °C until OD_600 nm_ 0.4–0.6 and then were induced by adding 0.1 mM isopropyl β-D-1-thiogalactopyranoside (IPTG). Subsequently, cultures were continued at 18 °C overnight. Cells were collected by centrifugation (10000 × g, 10 min) and washed with 50 mM Tris/HCl buffer, pH 8.0. Proteins were purified as previously described^36^,^38^.

### Enzyme activity assays

Enzyme activity assays of glucansucrases were performed as previously described^55^ in 25 mM sodium acetate/1 mM CaCl_2_ buffer, pH 5.0. The activity of all enzymes was measured at 35 °C with 30 nM enzymes and 100 mM sucrose. Samples of 25 μl were withdrawn every min over a 5 min period and inactivated with 2.5 μl 1 M NaOH. The activity was determined by measuring the amount of fructose released from sucrose. One unit of enzyme activity was defined as the release of 1 μmol of fructose per min.

### Thin-layer chromatography

Product mixtures from incubations of donor and acceptor substrates with glucansucrase enzymes were analyzed by TLC as previously described^37^.

### Production of α-glucans by wild-type and mutant enzymes

α-Glucans were produced by incubating wild-type and mutant enzymes (0.1 U/ml) with 100 mM sucrose at 35 °C in 25 mM sodium acetate/1 mM CaCl_2_ buffer, pH 5.0. The depletion of sucrose was followed by TLC analysis. After depletion of sucrose, enzymes were inactivated by incubating at 100 °C for 10 min. The amount of glucose in the incubation mixtures, which represents the hydrolysis of sucrose, was determined as previously described^44^ and the percentage of hydrolysis was calculated from the amount of glucose (hydrolysis) divided by the total amount of sucrose added in the incubation mixture. The percentage of transglycosylation was calculated by subtracting the percentage of hydrolysis from one. Polysaccharides were precipitated by adding two volumes of cold ethanol. The mixtures were incubated at 4 °C overnight and polysaccharides were collected by centrifugation (4 °C, 6000 × g, 20 min). The precipitated polysaccharides were washed again with two volumes of cold ethanol. Subsequently, polysaccharides were lyophilized for further structural analysis.

### High performance anion-exchange chromatography (HPAEC)

Samples of oligosaccharides were analyzed by HPAEC on a Dionex DX500 workstation (Dionex, Amsterdam, The Netherlands), equipped with an ED40 pulsed amperometric detection (PAD) system. The separation of oligosaccharides was carried out on a CarboPac PA-1 column (250 × 5 mm; Dionex) by using a linear gradient of 25–300 mM sodium acetate in 100 mM NaOH (1 ml/min).

### Pullulanase digestion of polysaccharides produced by wild-type and mutant enzymes

Samples of 4 mg polysaccharides were dissolved in 400 μl of 50 mM sodium acetate buffer, pH 5.0, and digested by adding 6 μl pullulanase M1 of *Klebsiella planticola* (Megazyme, Bray, Ireland) for 72 h. The completeness of the digestion of polysaccharides was checked by TLC analysis. Digestion mixtures were diluted 200 fold and analyzed by HPAEC-PAD using a linear gradient of 25–600 mM sodium acetate in 100 mM NaOH (1 ml/min). The amount of maltose in the digestion mixture was quantified by HPAEC using maltose as a standard. The percentage of maltose in the digestion mixture was calculated based on the amount of glucosyl units in relation to the total amount of glucosyl units in the added polysaccharide.

### Oligosaccharide synthesis by incubating wild-type and mutant enzymes with sucrose (donor substrate) and maltose or isomaltose or maltotriose or panose (acceptor substrate)

The wild-type and mutant enzymes (0.1 U/ml) were incubated with 100 mM sucrose and 100 mM maltose or 100 mM isomaltose or 100 mM maltotriose or 100 mM panose at 35 °C in 25 mM sodium acetate/1 mM CaCl_2_ buffer, pH 5.0. After depletion of sucrose, reactions were stopped by incubation at 100 °C for 10 min. Product mixtures were diluted 1000 fold and analyzed by HPAEC-PAD analysis. The oligosaccharides synthesized by incubating GTFA and GTFO with sucrose and maltose or isomaltose have been isolated and structurally characterized in our previous studies^37^,^38^. In the present study, the amount of remaining maltose, and the products panose, maltotriose and maltotetraose formed during incubations of 100 mM sucrose (donor substrate) and 100 mM maltose (acceptor substrate) was quantified using respective standards. Due to the lack of appropriate standards for glucosyl-(α1 → 4)-panose and glucosyl-(α1 → 6)-(α1 → 4)-panose, panose was used as standard for these compounds. The amount of remaining isomaltose, and the product isomaltotriose formed during incubations of 100 mM sucrose (donor substrate) and 100 mM isomaltose (acceptor substrate) was quantified using respective standards. Due to the lack of appropriate standards, the amount of produced isopanose, glucosyl-(α1 → 6)-isopanose [α-D-Glc*p*-(1 → 6)-α-D-Glc*p*-(1 → 4)-α-D-Glc*p*-(1 → 6)-D-Glc*p*] was estimated using panose as standard for these compounds. The yields of different oligosaccharides were calculated based on the percentage of maltose or isomaltose being converted to the respective oligosaccharides.

The oligosaccharides synthesized by incubation of GTFA-∆N and GTFO-∆N with 100 mM sucrose and 100 mM maltotriose or 100 mM panose were separated and isolated by HPAEC with a CarboPac PA-1 column (250 × 9 mm) using a linear gradient of 25–300 mM sodium acetate in 100 mM NaOH or isocratic conditions (3 ml/min). Collected fractions were neutralized immediately by adding 4 M acetic acid, and desalted on CarboGraph SPE columns (Alltech, Breda, The Netherlands) using acetonitrile:water = 2:3 as eluent. These samples were lyophilized and subjected to further structural analysis.

### Matrix-assisted laser-desorption ionization time-of-flight mass spectrometry

MALDI-TOF-MS experiments were performed on an Axima^TM^ Performance mass spectrometer (Shimadzu Kratos Inc., Manchester, UK), equipped with a nitrogen laser (337 nm, 3 ns pulse width) as previously described^37^.

### NMR spectroscopy

One-dimensional ^1^H NMR, 2D ^1^H-^1^H and 2D ^13^C-^1^H correlation spectra were recorded on a Varian Inova 500 Spectrometer (NMR Center, University of Groningen) at a probe temperature of 300 K or 310 K as previously described^37^. The respective anomeric signal peak areas of ^1^H NMR spectra of polysaccharides were integrated to calculate the ratio of different linkages. 2D ^1^H–^1^H TOCSY spectra were recorded with MLEV17 mixing sequences with 30, 60, and 150 ms spin-lock times. 2D ^1^H–^1^H ROESY spectra with a mixing time of 300 ms were recorded in 256 increments of 2000 complex data points with a spectral width of 5000 Hz. 2D ^13^C–^1^H HSQC spectra were recorded without decoupling during acquisition of the ^1^H free induction decay and with a spectral width of 5000 Hz in the t2 and 10,000 Hz in the t1 direction. All spectra were processed using MestReNova5.3 (Mestrelabs Research SL, Santiago de Compostela, Spain), using Whittaker Smoother baseline correction.

### Methylation analysis of polysaccharides produced by wild-type and mutant enzymes

Methylation analysis was performed as previously described^56^. Briefly, isolated polysaccharides (~5 mg) were permethylated using CH_3_I and solid NaOH in Me_2_SO_4_, hydrolyzed with 2 M trifluoroacetic acid (2 h, 120 °C), reduced with NaBD_4_ (2 h at room temperature, aqueous solution) and neutralized by adding 4 M acetic acid. Then the boric acid was co-evaporated with methanol. The mixture was acetylated using pyridine/acetic anhydride (1:1, v/v) for 30 min at 120 °C and then analyzed by GLC-EI-MS on a GCMS-QP2010 plus instrument (Shimadzu).

## Additional Information

**How to cite this article**: Meng, X. *et al.* Structural determinants of alternating (α1 → 4) and (α1 → 6) linkage specificity in reuteransucrase of *Lactobacillus reuteri. Sci. Rep.* **6**, 35261; doi: 10.1038/srep35261 (2016).

## Supplementary Material

Supplementary Information

## Figures and Tables

**Table 1:** Primer pairs used for site-directed mutagenesis of *gtfO*-ΔN.

Mutant	Mutations	Primer pairs (5′ → 3′)
A977Q	A977 → Q	A977Q-For: 5′-GCTACAAGGGTCAGGAGTTCCTGTTAGC-3′ A977Q-Rev: 5′-GCTAACAGGAACTCCTGACCCTTGTAGC-3′
lp977	^970^DGKGYKGA^977^ → KDQTYRGQ	A977LP1-For: 5′-TGATGGTAAGGGCTACCGAGGTCAAGAGTTCCTGTTAG-3′ A977LP1-Rev:5′-CTAACAGGAACTCTTGACCTCGGTAGCCCTTACCATCA-3′ A977LP2-For: 5′-GCCAATCAACATTAAGGATCAGACATACCGAGGTCAAG-3′ A977LP2-Rev: 5′-CTTGACCTCGGTATGTCTGATCCTTAATGTTGATTGGC-3′
SS-HA	S1065 → H/S1066 → A	SS-HA-For: 5′-CATCCTCGAAGACTGGAACCACGCGGATCCAAATTACTTTAAC-3′ SS-HA-Rev: 5′-GTTAAAGTAATTTGGATCCGCGTGGTTCCAGTCTTCGAGGATG-3′
α4	^1083^VSLKGA^1088^ → DTIKNS	V1083LP-For: 5′-GCTAACGATGGATGATACTATTAAGAATTCCCTTAACCATGGAC-3′ V1083LP-Rev: 5′-GTCCATGGTTAAGGGAATTCTTAATAGTATCATCCATCGTTAGC-3′
V1083D	V1083 → D	V1083D-For: 5′-GCTAACGATGGATGATTCGCTTAAGGGC-3′ V1083D-Rev: 5′-GCCCTTAAGCGAATCATCCATCGTTAGC-3′
G1087N	G1087 → N	G1087N-For: 5′-GGATGTTTCGCTTAAGAACGCCCTTAACCATGGAC-3′ G1087N-Rev: 5′-GTCCATGGTTAAGGGCGTTCTTAAGCGAAACATCC-3′
V1083D/G1087N	V1083 → D/G1087 → N	V1083DG1087N-For: 5′-CTAACGATGGATGATTCGCTTAAGAACGCCCTTAACC-3′ V1083DG1087N-Rev: 5′-GGTTAAGGGCGTTCTTAAGCGAATCATCCATCGTTAG-3′

The mutated nucleotides are underlined.

**Table 2:** Specific activity and hydrolysis/transglycosylation ratio of wild-type GTFO-∆N and GTFA-∆N enzymes, and of GTFO-derived mutants, at 100 mM sucrose, and methylation, ^1^H NMR and pullulanase digestion analysis of the polysaccharides produced.

Enzyme	Activity^a^ (U/mg)	Hydrolysis/trans-glycosylation (%)	Chemical shift^c^(%)		Methylation analysis^b^(%)				Maltose^d^
			(α1 → 6)	(α1 → 4)	Glc*p*(1 →	→ 4)Glc*p*(1 →	→6)Glc*p*(1→	→4,6)Glc*p*(1→	
GTFA-ΔN	22.7 ± 1.8	9/91	42	58	13	45	27	15	45.9 ± 2.2
GTFO-ΔN	13.7 ± 0.9	43/57	21	79	11	69	9	11	8.7 ± 1.3
GTFO-ΔN A977Q	14.4 ± 1.2	35/65	31	69	16	51	18	15	24.9 ± 3.2
GTFO-ΔN lp977	1.5 ± 0.3	35/65	33	67	14	53	18	15	25.5 ± 2.0
GTFO-ΔN SS-HA	11.0 ± 1.5	44/56	24	76	12	68	10	10	8.3 ± 1.0
GTFO-ΔN α4	13.3 ± 2.6	37/63	36	64	19	43	21	18	28.1 ± 2.4
GTFO-ΔN A977Q/α4	12.2 ± 1.2	33/67	39	61	18	42	22	18	35.8 ± 3.0
GTFO-ΔN lp977/α4	1.2 ± 0.3	30/70	40	60	17	44	22	17	31.6 ± 3.1
GTFO-ΔN SS-HA/α4	13.4 ± 0.9	37/63	36	64	16	45	22	17	29.1 ± 3.5
GTFO-ΔN lp977/SS-HA	0.3 ± 0.08	36/64	31	69	13	56	19	12	27.0 ± 3.3
GTFO-ΔN lp977/SS-HA/α4	1.9 ± 0.4	30/70	40	60	17	44	23	16	34.1 ± 2.8
GTFO-ΔN A977Q/SS-HA/α4	15.8 ± 2.2	31/69	39	61	15	45	23	17	35.4 ± 3.6
GTFO-ΔN A977Q/V1083D	17.7 ± 1.5	34/66	36	64	18	42	22	18	28.8 ± 2.5
GTFO-ΔN A977Q/G1087N	29.0 ± 3.0	31/69	34	66	16	47	20	17	25.2 ± 2.1
GTFO-ΔN A977Q/V1083D/G1087N	21.9 ± 1.2	30/70	38	62	17	42	22	19	30.3 ± 2.8

^a^Activity measured at 100 mM sucrose at 35 °C. ^b^The ratios of the different glucosyl substitutions are shown in molar percentage based on the intensities from GLC. ^c^The ratios of integration of the surface areas of the (α1 → 6) linkage signal at 4.96 ppm and the (α1 → 4) linkage signal at 5.36 ppm in the ^1^H NMR spectra are displayed. ^d^The percentage of maltose in the pullulanase M1 digestion product mixtures of polysaccharides produced by the respective enzymes based on the glucosyl unit.

**Table 3:** Product distribution determined after incubation of wild-type GTFO-∆N and GTFA-∆N enzymes, and of GTFO-derived mutants, with 100 mM sucrose and 100 mM maltose.

Enzyme	Oligosaccharide yield (%)	Panose (%)	Maltotriose	Glucosyl-(α1 → 4)-panose	Glucosyl-(α1 → 6)- (α1 → 4)-panose	Maltotetraose
GTFA-ΔN	69.9 ± 3.4	51.1 ± 3.5	3.3 ± 0.4	8.9 ± 0.9	6.5 ± 0.7	ND
GTFO-ΔN	60.7 ± 2.8	29.5 ± 2.7	12.1 ± 1.1	14.2 ± 0.8	ND	4.9 ± 0.8
GTFO-ΔN A977Q	69.1 ± 3.7	44.3 ± 3.1	7.0 ± 0.9	14.8 ± 1.1	0.5 ± 0.2	2.5 ± 0.5
GTFO-ΔN lp977	68.0 ± 3.8	43.2 ± 2.9	6.1 ± 1.0	15.1 ± 1.6	0.5 ± 0.2	3.1 ± 0.5
GTFO-ΔN SS-HA	59.7 ± 2.1	29.5 ± 2.3	11.9 ± 1.2	14.0 ± 1.1	ND	4.2 ± 0.6
GTFO-ΔN α4	55.6 ± 3.0	25.8 ± 2.0	10.7 ± 1.4	9.9 ± 1.2	5.7 ± 0.8	3.6 ± 0.5
GTFO-ΔN A977Q/α4	65.7 ± 2.1	42.4 ± 2.4	5.8 ± 0.6	9.2 ± 1.1	6.2 ± 1.2	2.0 ± 0.5
GTFO-ΔN lp977/α4	67.8 ± 6.3	43.8 ± 3.8	5.5 ± 0.8	9.4 ± 1.2	6.7 ± 1.0	2.4 ± 0.4
GTFO-ΔN SS-HA/α4	58.0 ± 1.5	26.6 ± 1.7	11.4 ± 1.5	12.6 ± 1.5	2.1 ± 0.4	5.2 ± 0.5
GTFO-ΔN lp977/SS-HA	69.0 ± 3.7	43.0 ± 4.3	6.4 ± 1.0	16.1 ± 1.6	ND	3.4 ± 0.6
GTFO-ΔN lp977/SS-HA/α4	68.7 ± 3.5	40.2 ± 2.1	7.2 ± 1.1	14.2 ± 0.9	3.8 ± 1.1	3.3 ± 1.2
GTFO-ΔN A977Q/SS-HA/α4	65.8 ± 2.7	39.2 ± 2.7	7.1 ± 1.3	13.3 ± 1.3	3.2 ± 0.6	3.0 ± 0.4
GTFO-ΔN A977Q/V1083D	69.6 ± 4.5	44.8 ± 2.9	5.7 ± 0.9	13.2 ± 0.9	3.5 ± 0.4	2.3 ± 0.5
GTFO-ΔN A977Q/G1087N	70.7 ± 4.9	41.4 ± 3.2	7.0 ± 0.7	17.5 ± 1.4	0.5 ± 0.2	4.2 ± 0.5
GTFO-ΔN A977Q/V1083D/G1087N	68.4 ± 5.4	43.4 ± 2.8	5.7 ± 0.8	10.1 ± 0.9	6.7 ± 0.8	2.4 ± 0.4

^a^After depletion of sucrose, the amounts of individual oligosaccharides formed were determined by HPAEC-PAD and expressed as the percentage of maltose initially present in the incubation. ND: not detected.

**Table 4:** Product distribution determined after incubation of wild-type GTFO-∆N and GTFA-∆N enzymes, and of GTFO-derived mutants, with 100 mM sucrose and 100 mM isomaltose.

Enzyme	Oligosaccharide yields (%)	Isomaltotriose (%)	Isopanose (%)	Glucosyl-(α1 → 6) Isopanose (%)
GTFA-ΔN	32.0 ± 0.6	3.1 ± 0.4	14.5 ± 1.8	14.4 ± 1.9
GTFO-ΔN	40.6 ± 3.3	1.4 ± 0.4	36.3 ± 2.2	2.9 ± 0.8
GTFO-ΔN A977Q	36.1 ± 2.1	2.8 ± 0.4	29.5 ± 2.2	3.8 ± 0.5
GTFO-ΔN lp977	36.2 ± 1.3	2.6 ± 0.3	29.2 ± 2.0	4.5 ± 0.4
GTFO-ΔN SS-HA	38.7 ± 2.5	1.1 ± 0.2	35.3 ± 1.7	2.3 ± 0.5
GTFO-ΔN α4	35.8 ± 2.1	1.3 ± 0.2	24.7 ± 1.7	9.8 ± 0.8
GTFO-ΔN A977Q/α4	30.5 ± 1.4	2.3 ± 0.2	15.9 ± 1.1	12.3 ± 1.2
GTFO-ΔN lp977/α4	30.1 ± 2.5	1.9 ± 0.3	14.7 ± 1.6	13.5 ± 1.1
GTFO-ΔN SS-HA/α4	42.5 ± 3.0	1.0 ± 0.3	34.7 ± 2.7	6.7 ± 0.8
GTFO-ΔN lp977/SS-HA	35.9 ± 0.9	2.2 ± 0.4	28.1 ± 2.4	5.6 ± 1.2
GTFO-ΔN lp977/SS-HA/α4	37.7 ± 3.9	1.5 ± 0.2	25.4 ± 2.1	10.8 ± 1.6
GTFO-ΔN A977Q/SS-HA/α4	36.8 ± 3.0	1.8 ± 0.3	26.4 ± 1.9	8.6 ± 0.8
GTFO-ΔN A977Q/V1083D	35.4 ± 2.8	2.2 ± 0.3	24.5 ± 1.8	8.7 ± 0.9
GTFO-ΔN A977Q/G1087N	37.2 ± 1.9	2.2 ± 0.3	32.0 ± 2.8	3.1 ± 0.6
GTFO-ΔN A977Q/V1083D/G1087N	31.5 ± 2.6	2.1 ± 0.3	17.2 ± 1.7	12.2 ± 1.6

^a^After depletion of sucrose, the amount of individual oligosaccharides formed were determined by HPAEC-PAD and expressed as the percentage of isomaltose initially present in the incubation.

## References

[b1] Lindgren S. E. & Dobrogosz W. J. Antagonistic activities of lactic acid bacteria in food and feed fermentations. FEMS Microbiol. Rev. 7, 149–163 (1990). 2125429 10.1111/j.1574-6968.1990.tb04885.x

[b2] Badel S., Bernardi T. & Michaud P. New perspectives for *Lactobacilli* exopolysaccharides. Biotechnol. Adv. 29, 54–66 (2011). 20807563 10.1016/j.biotechadv.2010.08.011

[b3] Bounaix M. S. *et al.* Biodiversity of exopolysaccharides produced from sucrose by sourdough lactic acid bacteria. J. Agric. Food Chem. 57, 10889–10897 (2009). 19848387 10.1021/jf902068t

[b4] Galle S. & Arendt E. K. Exopolysaccharides from sourdough lactic acid bacteria. Crit. Rev. Food Sci. Nutr. 54, 891–901 (2014). 24499068 10.1080/10408398.2011.617474

[b5] Monsan P. *et al.* Homopolysaccharides from lactic acid bacteria. Int. Dairy J. 11, 675–685 (2001).

[b6] Naessens M., Cerdobbel A., Soetaert W. & Vandamme E. J. Leuconostoc dextransucrase and dextran: production, properties and applications. J. Chem. Technol. Biotechnol. 80, 845–860 (2005).

[b7] Patel S., Majumder A. & Goyal A. Potentials of exopolysaccharides from lactic acid bacteria. Indian J. Microbiol. 52, 3–12 (2012). 23449986 10.1007/s12088-011-0148-8 PMC3298600

[b8] Monchois V., Willemot R. M. & Monsan P. Glucansucrases: mechanism of action and structure-function relationships. FEMS Microbiol. Rev. 23, 131–151 (1999). 10234842 10.1111/j.1574-6976.1999.tb00394.x

[b9] van Hijum S. A., Kralj S., Ozimek L. K., Dijkhuizen L. & van Geel-Schutten I. G. Structure-function relationships of glucansucrase and fructansucrase enzymes from lactic acid bacteria. Microbiol. Mol. Biol. Rev. 70, 157–176 (2006). 16524921 10.1128/MMBR.70.1.157-176.2006 PMC1393251

[b10] Leemhuis H. *et al.* Glucansucrases: three-dimensional structures, reactions, mechanism, α-glucan analysis and their implications in biotechnology and food applications. J. Biotechnol. 163, 250–272 (2013). 22796091 10.1016/j.jbiotec.2012.06.037

[b11] Korakli M. & Vogel R. F. Structure/function relationship of homopolysaccharide producing glycansucrases and therapeutic potential of their synthesised glycans. Appl. Microbiol. Biotechnol. 71, 790–803 (2006). 16724190 10.1007/s00253-006-0469-4

[b12] André I., Potocki-Vèronése G., Morel S., Monsan P. & Remaud-Siméon M. Sucrose-utilizing transglucosidases for biocatalysis. Top. Curr. Chem. 294, 25–48 (2010). 21626747 10.1007/128_2010_52

[b13] Lombard V., Golaconda Ramulu H., Drula E., Coutinho P. M. & Henrissat B. The carbohydrate-active enzymes database (CAZy) in 2013. Nucleic Acids Res. 42, D490–D495 (2014). 24270786 10.1093/nar/gkt1178 PMC3965031

[b14] MacGregor E. A., Jespersen H. M. & Svensson B. A circularly permuted α-amylase-type α/β-barrel structure in glucan-synthesizing glucosyltransferases. FEBS Lett. 378, 263–266 (1996). 8557114 10.1016/0014-5793(95)01428-4

[b15] Kralj S., van Geel-Schutten I. G., Faber E. J., van der Maarel M. J. E. C. & Dijkhuizen L. Rational transformation of *Lactobacillus reuteri* 121 reuteransucrase into a dextransucrase. Biochemistry 44, 9206–9216 (2005). 15966745 10.1021/bi050447q

[b16] Vujičić-Žagar A. *et al.* Crystal structure of a 117 kDa glucansucrase fragment provides insight into evolution and product specificity of GH70 enzymes. Proc. Natl. Acad. Sci. USA 107, 21406–21411 (2010). 21118988 10.1073/pnas.1007531107 PMC3003066

[b17] Hamada S. & Slade H. D. Biology, immunology, and cariogenicity of *Streptococcus mutans*. Microbiol. Rev. 44, 331–384 (1980). 6446023 10.1128/mr.44.2.331-384.1980 PMC373181

[b18] Gibbons R. J. Formation and significance of bacterial polysaccharides in caries etiology. Caries Res. 2, 164–171 (1968). 5248533 10.1159/000259554

[b19] Ooshima T. *et al.* Contributions of three glucosyltransferases to sucrose-dependent adherence of *Streptococcus mutans*. J. Dent. Res. 80, 1672–1677 (2001). 11597030 10.1177/00220345010800071401

[b20] Bowen W. H. & Koo H. Biology of *Streptococcus mutans*-derived glucosyltransferases: role in extracellular matrix formation of cariogenic biofilms. Caries Res. 45, 69–86 (2011). 10.1159/000324598 PMC3068567 21346355

[b21] Argüello-Morales M. A. *et al.* Sequence analysis of the gene encoding alternansucrase, a sucrose glucosyltransferase from *Leuconostoc mesenteroides* NRRL B-1355. FEMS Microbiol. Lett. 182, 81–85 (2000). 10612736 10.1111/j.1574-6968.2000.tb08878.x

[b22] Côté G. L. & Robyt J. F. Isolation and partial characterization of an extracellular glucansucrase from *Leuconostoc mesenteroides* NRRL B-1355 that synthesizes an alternating (1tion of an extracellul*Carbohydr*. Res. 101, 57–74 (1982). 10.1016/s0008-6215(00)80795-8 7060056

[b23] Côté G. L. & Sheng S. Penta-, hexa-, and heptasaccharide acceptor products of alternansucrase. Carbohydr. Res. 341, 2066–2072 (2006). 16716279 10.1016/j.carres.2006.04.044

[b24] Smith M. R., Zahnley J. & Goodman N. Glucosyltransferase mutants of *Leuconostoc mesenteroides* NRRL B-1355. Appl. Environ. Microbiol. 60, 2723–2731 (1994). 16349346 10.1128/aem.60.8.2723-2731.1994 PMC201715

[b25] Côté G. L. Acceptor products of alternansucrase with gentiobiose. Production of novel oligosaccharides for food and feed and elimination of bitterness. Carbohydr. Res. 344, 187–190 (2009). 19056079 10.1016/j.carres.2008.10.017

[b26] Côté G. L., Dunlap C. A. & Vermillion K. E. Glucosylation of raffinose via alternansucrase acceptor reactions. Carbohydr. Res. 344, 1951–1959 (2009). 19596226 10.1016/j.carres.2009.06.023

[b27] Côté G. L., Dunlap C. A., Appell M. & Momany F. A. Alternansucrase acceptor reactions with D-tagatose and L-glucose. Carbohydr. Res. 340, 257–262 (2005). 15639245 10.1016/j.carres.2004.11.013

[b28] Côté G. L. & Dunlap C. A. Alternansucrase acceptor reactions with methyl hexopyranosides. Carbohydr. Res. 338, 1961–1967 (2003). 14499572 10.1016/s0008-6215(03)00324-0

[b29] Hernandez-Hernandez O., Côté G. L., Kolida S., Rastall R. A. & Sanz M. L. *In vitro* fermentation of alternansucrase raffinose-derived oligosaccharides by human gut bacteria. J. Agric. Food Chem. 59, 10901–10906 (2011). 21913653 10.1021/jf202466s

[b30] Holt S. M., Miller-Fosmore C. M. & Côté G. L. Growth of various intestinal bacteria on alternansucrase-derived oligosaccharides. Lett. Appl. Microbiol. 40, 385–390 (2005). 15836744 10.1111/j.1472-765X.2005.01681.x

[b31] Holt S. M., Teresi J. M. & Côté G. L. Influence of alternansucrase-derived oligosaccharides and other carbohydrates on alpha-galactosidase and alpha-glucosidase activity in Bifidobacterium adolescentis. Lett. Appl. Microbiol. 46, 73–79 (2008). 17971098 10.1111/j.1472-765X.2007.02266.x

[b32] Sanz M. L., Côté G. L., Gibson G. R. & Rastall R. A. Prebiotic properties of alternansucrase maltose-acceptor oligosaccharides. J. Agric. Food Chem. 53, 5911–5916 (2005). 16028973 10.1021/jf050344e

[b33] van Geel-Schutten G. H., Flesch F., ten Brink B., Smith M. R. & Dijkhuizen L. Screening and characterization of *Lactobacillus* strains producing large amounts of exopolysaccharides. Appl. Microbiol. Biotechnol. 50, 697–703 (1998).

[b34] van Leeuwen S. S. *et al.* Structural analysis of the α-D-glucan (EPS35-5) produced by the *Lactobacillus reuteri* strain 35-5 glucansucrase GTFA enzyme. Carbohydr. Res. 343, 1251–1265 (2008). 18313039 10.1016/j.carres.2008.01.044

[b35] Kralj S. *et al.* Molecular characterization of a novel glucosyltransferase from *Lactobacillus reuteri* strain 121 synthesizing a unique, highly branched glucan with. & Dijkh α-(1 → 6) glucosidic bonds. Appl. Environ. Microbiol. 68, 4283–4291 (2002). 12200277 10.1128/AEM.68.9.4283-4291.2002 PMC124066

[b36] Kralj S., van Geel-Schutten G. H., van der Maarel M. J. E. C. & Dijkhuizen L. Biochemical and molecular characterization of *Lactobacillus reuteri* 121 reuteransucrase. Microbiology 150, 2099–2112 (2004). 15256553 10.1099/mic.0.27105-0

[b37] Dobruchowska J. M. *et al.* Gluco-oligomers initially formed by the reuteransucrase enzyme of *Lactobacillus reuteri* 121 incubated with sucrose and malto-oligosaccharides. Glycobiology 23, 1084–1096 (2013). 23804502 10.1093/glycob/cwt048

[b38] Kralj S., Stripling E., Sanders P., van Geel-Schutten G. H. & Dijkhuizen L. Highly hydrolytic reuteransucrase from probiotic *Lactobacillus reuteri* strain ATCC 55730. Appl. Environ. Microbiol. 71, 3942–3950 (2005). 16000808 10.1128/AEM.71.7.3942-3950.2005 PMC1169070

[b39] Côté G. L. & Skory C. D. Effects of mutations at threonine-654 on the insoluble glucan synthesized by *Leuconostoc mesenteroides* NRRL B-1118 glucansucrase. Appl. Microbiol. Biotechnol. 98, 6651–6658 (2014). 24682484 10.1007/s00253-014-5622-x

[b40] Hellmuth H. *et al.* Engineering the glucansucrase GTFR enzyme reaction and glycosidic bond specificity: toward tailor-made polymer and oligosaccharide products. Biochemistry 47, 6678–6684 (2008). 18512955 10.1021/bi800563r

[b41] Leemhuis H., Pijning T., Dobruchowska J. M., Dijkstra B. W. & Dijkhuizen L. Glycosidic bond specificity of glucansucrases: on the role of acceptor substrate binding residues. Biocatal. Biotransform. 30, 366–376 (2012).

[b42] Moulis C. *et al.* Understanding the polymerization mechanism of glycoside-hydrolase family 70 glucansucrases. J. Biol. Chem. 281, 31254–31267 (2006). 16864576 10.1074/jbc.M604850200

[b43] van Leeuwen S. S. *et al.* Structural characterization of bioengineered α-D-glucans produced by mutant glucansucrase GTF180 enzymes of *Lactobacillus reuteri* strain 180. Biomacromolecules 10, 580–588 (2009). 19186951 10.1021/bm801240r

[b44] Meng X. *et al.* Residue Leu^940^ has a crucial role in the linkage and reaction specificity of the glucansucrase GTF180 of the probiotic bacterium *Lactobacillus reuteri* 180. J. Biol. Chem. 289, 32773–32782 (2014). 25288798 10.1074/jbc.M114.602524 PMC4239627

[b45] Meng X., Pijning T., Dobruchowska J. M., Gerwig G. J. & Dijkhuizen L. Characterization of the functional roles of amino acid residues in acceptor binding subsite +1 in the active site of the glucansucrase GTF180 enzyme of *Lactobacillus reuteri* 180. J. Biol. Chem. 290, 30131–30141 (2015). 26507662 10.1074/jbc.M115.687558 PMC4705985

[b46] Janecek S. How many conserved sequence regions are there in the α-amylase family ? Biologia 57, 29–41 (2002).

[b47] Gangoiti J., Pijning T. & Dijkhuizen L. The Exiguobacterium sibiricum 255-15 GtfC Enzyme Represents a Novel Glycoside Hydrolase 70 Subfamily of 4,6-α-Glucanotransferase Enzymes. Appl. Environ. Microbiol. 82, 756–766 (2016). 10.1128/AEM.03420-15 PMC4711130 26590275

[b48] Pijning T., Vujičić-Žagar A., Kralj S., Dijkhuizen L. & Dijkstra B. W. Structure of the α-1,6/α-1,4-specific glucansucrase GTFA from Lactobacillus reuteri 121. Acta Crystallogr. Sect. F Struct. Biol. Cryst. Commun. 68, 1448–1454 (2012). 10.1107/S1744309112044168 PMC3509963 23192022

[b49] van Leeuwen S. S., Leeflang B. R., Gerwig G. J. & Kamerling J. P. Development of a ^1^H NMR structural-reporter-group concept for the primary structural characterisation of α-D-glucans. Carbohydr. Res. 343, 1114–1119 (2008). 18314096 10.1016/j.carres.2008.01.043

[b50] Irague R. *et al.* Combinatorial engineering of dextransucrase specificity. PloS one 8, e77837 (2013). 24204991 10.1371/journal.pone.0077837 PMC3799614

[b51] Meng X., Dobruchowska J. M., Pijning T., Gerwig G. J. & Dijkhuizen L. Synthesis of new hyperbranched α-glucans from sucrose by *Lactobacillus reuteri 180* glucansucrase mutants. J. Agric. Food Chem. 64, 433–442 (2016). 26688101 10.1021/acs.jafc.5b05161

[b52] Robert X. & Gouet P. Deciphering key features in protein structures with the new ENDscript server. Nucleic Acids Res. 42, W320–W324 (2014). 24753421 10.1093/nar/gku316 PMC4086106

[b53] Pijning T., Vujičić-Žagar A., Kralj S., Dijkhuizen L. & Dijkstra B. W. Structure of the α-1,6/α-1,4-specific glucansucrase GTFA from *Lactobacillus reuteri* 121. Acta Crystallogr. Sect. F Struct. Biol. Cryst. Commun. 68, 1448–1454 (2012). 10.1107/S1744309112044168 PMC3509963 23192022

[b54] Kelley L. A., Mezulis S., Yates C. M., Wass M. N. & Sternberg M. J. The Phyre2 web portal for protein modeling, prediction and analysis. Nat. Protoc. 10, 845–858 (2015). 25950237 10.1038/nprot.2015.053 PMC5298202

[b55] Van Geel-Schutten G. H. *et al.* Biochemical and structural characterization of the glucan and fructan exopolysaccharides synthesized by the *Lactobacillus reuteri* wild-type strain and by mutant strains. Appl. Environ. Microbiol. 65, 3008–3014 (1999). 10388696 10.1128/aem.65.7.3008-3014.1999 PMC91449

[b56] van Leeuwen S. S. *et al.* Structural analysis of the α-D-glucan (EPS180) produced by the *Lactobacillus reuteri* strain 180 glucansucrase GTF180 enzyme. Carbohydr. Res. 343, 1237–1250 (2008). 18313038 10.1016/j.carres.2008.01.042

