# Supplementary material for: Structural determinants of alternating (α1 → 4) and (α1 → 6) linkage specificity in reuteransucrase of Lactobacillus reuteri
Source: Sci Rep. 2016 Oct 17;6:35261. doi: 10.1038/srep35261 (PMC5066211; doi:10.1038/srep35261)
Supplement: Supplementary Information [file srep35261-s1.pdf]

Supplementary Information

Structural determinants of alternating (α 1→4) and (α 1→6) linkage specificity in reuteransucrase of *Lactobacillus reuteri*

XiangfengMeng[1], Tjaard Pijning[2], Justyna M. Dobruchowska[1], Huifang Yin[1], Gerrit J. Gerwig[1]and Lubbert Dijkhuizen[1,]*

[1]Microbial Physiology, Groningen Biomolecular Sciences and Biotechnology Institute (GBB), University of Groningen, Nijenborgh 7, 9747 AG Groningen, The Netherlands,

[2]Biophysical Chemistry, Groningen Biomolecular Sciences and Biotechnology Institute (GBB), University of Groningen, Nijenborgh 7, 9747 AG Groningen, The Netherlands

*Corresponding author: l.dijkhuizen@rug.nl.

Structural characterization of oligosaccharide 7 from the incubation of 100 mM sucrose and 100 mM panose with GTFO-ΔN

As an example for the interpretation of the various ^{1}H and ^{13}C NMR assignments, the 1D ^{1}H and 2D (TOCSY, ROESY and HSQC) NMR spectra of oligosaccharide 7 (*m/z*, 829.5, $[M+H]^+$ according to MALDI-TOF-MS), are presented in Figure S2. The ^{1}H NMR spectrum showed 6 anomeric signals, which were used as the starting point for the assignment of the chemical shift of all nonanomeric protons in the 2D TOCSY spectrum. The ^{1}H anomeric signals at δ 5.230 (α) and δ 4.656 (β), and the set of their nonanomeric protons (Table S1) showed the presence of a reducing -(1→4)-α-D-Glc*p* **R** unit. The anomeric signal **B** (δ 4.977) revealed the presence of an (α1→6) linkage. Its set of anomeric protons (H-2, H-3, H-4, H-5, H-6a and H-6b at δ 3.59, 4.01, 3.66, 3.86, 3.89 and 3.83 respectively) correspond to that of internal -(1→4)-α-D-Glc*p*-(1→6)- unit. The set of chemical shifts of **A** H-1 H-2, H-3, H-4, H-5, H-6a and H-6b at δ 5.349, 3.64, 3.97, 3.67, 4.03, 3.87 and 3.95, respectively, revealed a -(1→4,6)-α-D-Glc*p*-(1→6)- unit. The 4- and 4,6-substitution of residues **B** and **A** are further supported by their ^{13}C chemical shifs (deduced from HSQC measurements, Figure S2): **B** C-4 at δ 78.4, **A** C-4 and **A** C-6 at δ 78.5 and δ 68.1, respectively. The anomeric signals of **C** (δ 5.360) and **C'** (δ 5.368) strongly overlap and they only show slight different chemical shifts of H-1, H-2 and H-4. Nevertheless, the set of their proton chemical shifts (Table S1) clearly assigned both of them as terminal α-D-Glc*p*-(1→4)- units. In the ROESY spectrum (Figure S2), inter-residual cross-peaks were observed between **A** H-1 and **Rα** H-4, **Rβ** H-4 (**A**1→4**R**), **B** H-1 and **A** H-6a (**B**1→6**A**), and **C/C'** H-1 and **A**4, **B**4 (**C/C'1**→4**A** and **C/C'1**→4**B**). Due to the highly overlapping signal of C and C', they can be exchanged, however, resulting in the same oligosaccharide structure. Taken together, these data lead to the characterization of oligosaccharide 7 as α-D-Glc*p*-(1→4)-[α-D-Glc*p*-(1→4)-α-D-Glc*p*-(1→6)]-D-Glc*p*-(1→4)-D-Glc*p*.

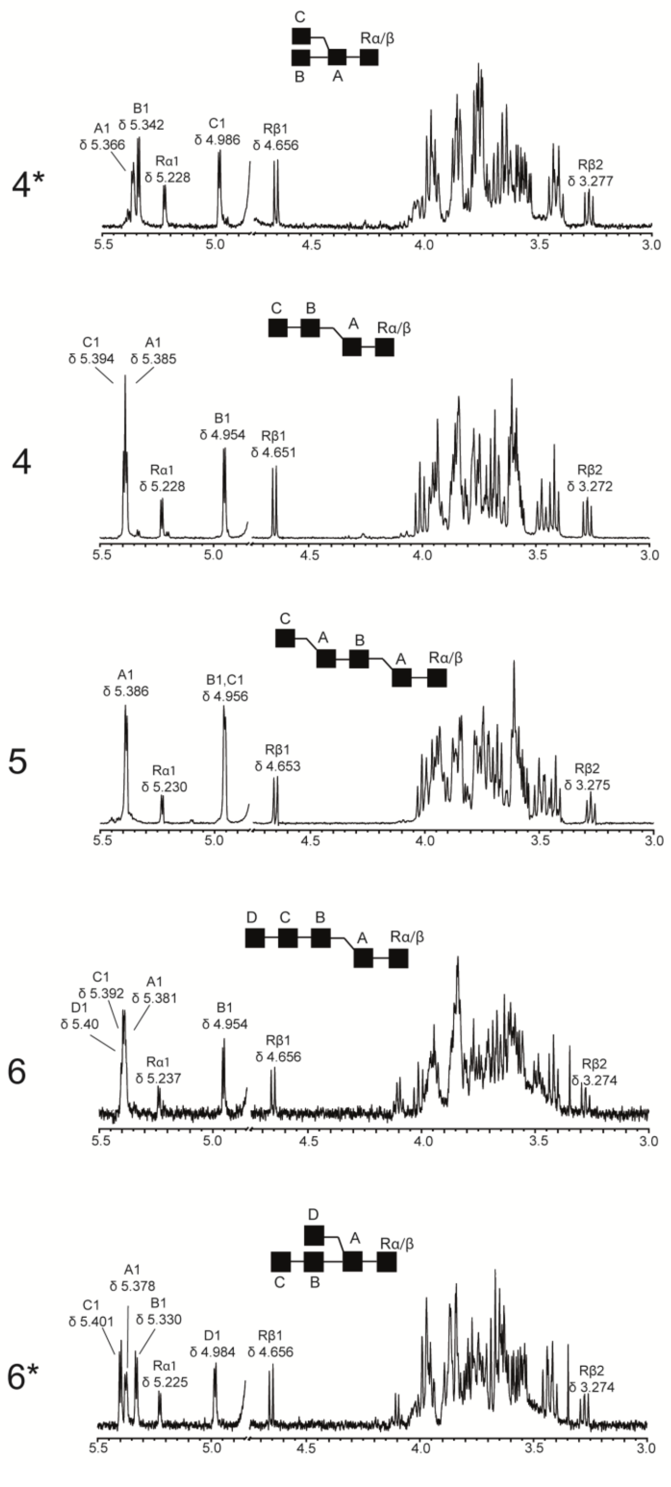

Figure S1. ^{1}H NMR spectra (300K) of oligosaccharides produced by incubation of sucrose (100 mM) and panose (100 mM) with the GTFO-ΔN enzyme of *Lactobacillus reuteri* ATCC 55730.

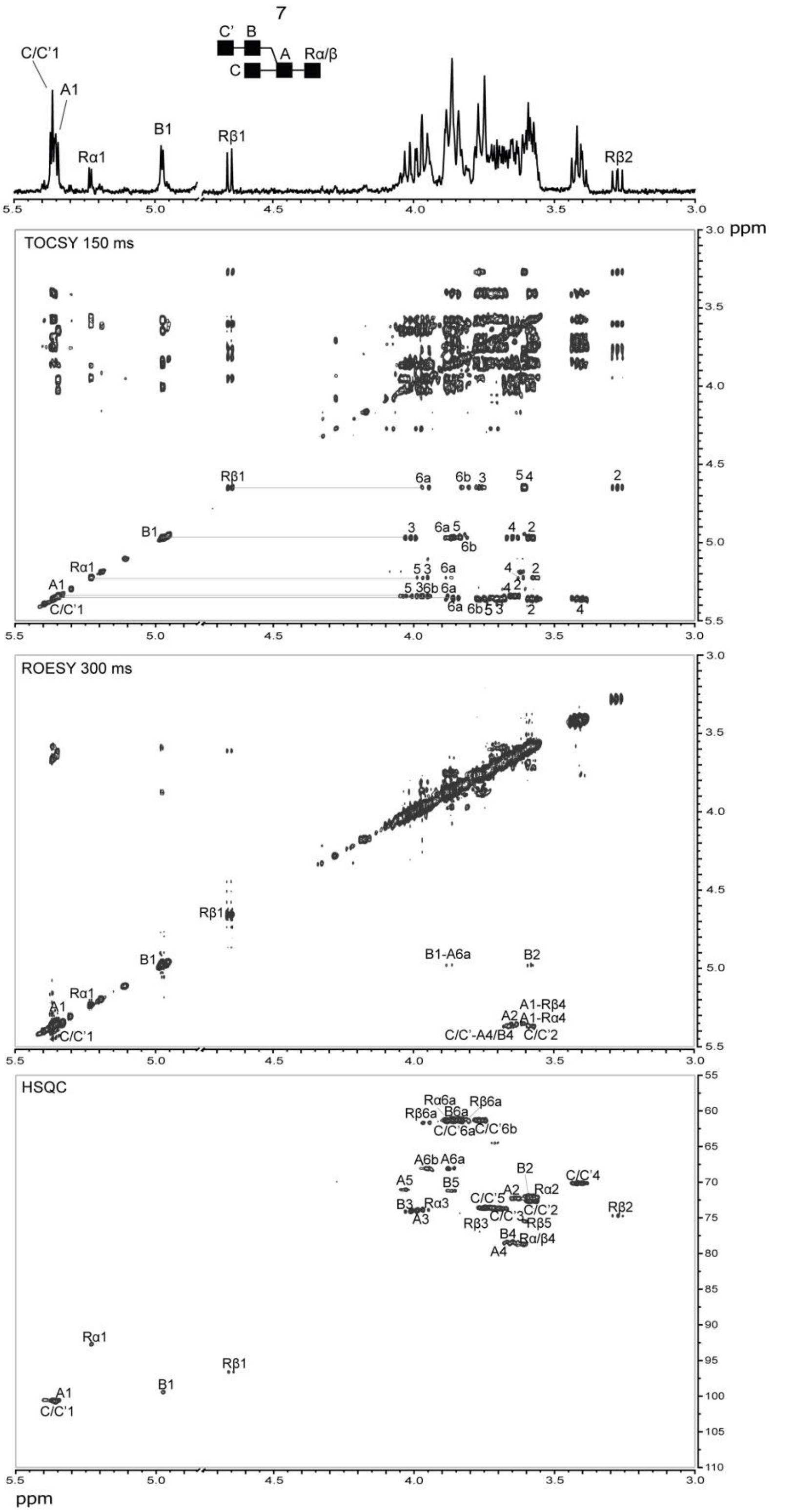

Figure S2. 1D ^{1}H NMR, TOCSY (150 ms), ROESY (300 ms) and HSQC spectra of α-D-Glc*p*-(1→4)-[α-D-Glc*p*-(1→4)-α-D-Glc*p*-(1→6)]-D-Glc*p*-(1→4)-D-Glc*p* (compound 7 in Figure 5).

4

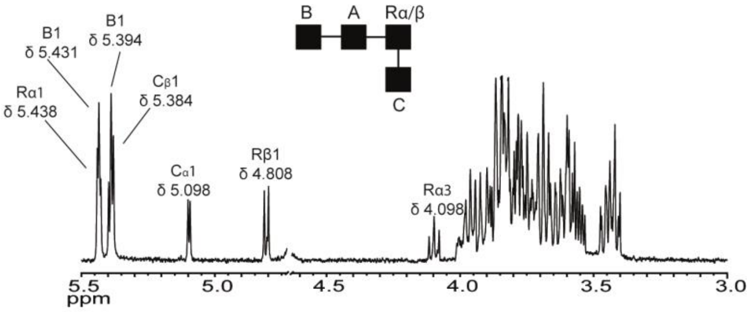

5

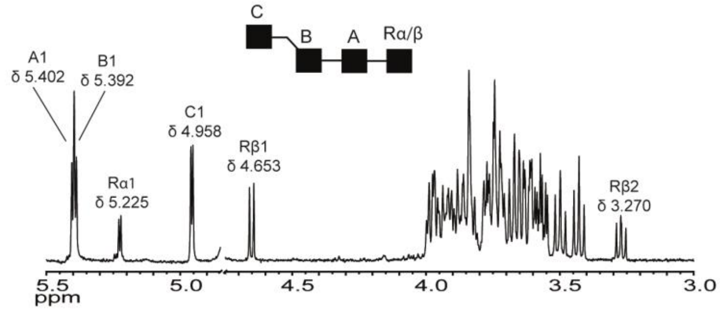

7

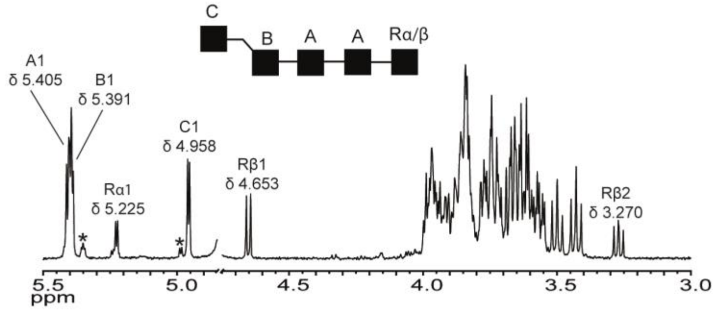

Figure S3. ^{1}H NMR spectra of oligosaccharides produced by incubation of sucrose (100 mM) and maltotriose (100 mM) with the GTFA-ΔN enzyme of *Lactobacillus reuteri* 121. The ^{1}H NMR spectrum of 4 was recorded at 310K while those of 5 and 7 were recorded at 300K.

Table S1. ^{1}H and ^{13}C chemical shifts[a] (D_2O, 300K) of Glc residues present in oligosaccharides (6, 6* and 7, Figure 6) formed by incubation of sucrose (donor substrate) and panose (acceptor substrate) with the GTFO-ΔN enzyme of *Lactobacillus reuteri* ATCC 55730.

Compound	H-1<br>C-1	H-2<br>C-2	H-3<br>C-3	H-4<br>C-4	H-5<br>C-5	H-6a<br>C-6	H-6b
6							
-(1→4)-α-D-Glc*p* (**Rα**)	5.230	3.57	3.98	3.63	3.95	3.88	3.83
-(1→4)-α-D-Glc*p* (**Rβ**)	4.656	3.27	3.77	3.63	3.65	3.95	3.79
-(1→6)-α-D-Glc*p*-(1→4)- (**A**)	5.381	3.61	3.68	3.48	3.94	3.76	3.98
-(1→4)-α-D-Glc*p*-(1→6)- (**B**)	4.954	3.60	4.00	3.65	3.85	3.89	3.82
-(1→4)-α-D-Glc*p*-(1→4)- (**C**)	5.392	3.62	3.96	3.66	3.84	3.87	3.82
α-D-Glc*p*-(1→4)- (**D**)	5.400	3.58	3.68	3.42	3.75	3.84	3.77
6*							
-(1→4)-α-D-Glc*p* (**Rα**)	5.225	3.57	3.98	3.63	3.95	3.88	3.83
-(1→4)-α-D-Glc*p* (**Rβ**)	4.656	3.27	3.77	3.63	3.65	3.95	3.79
-(1→4,6)-α-D-Glc*p*-(1→4)- (**A**)	5.378	3.64	3.97	3.67	4.01	3.85	4.03
-(1→4)-α-D-Glc*p*-(1→4)- (**B**)	5.330	3.62	3.97	3.65	3.84	3.88	3.81
α-D-Glc*p*-(1→4)- (**C**)	5.401	3.58	3.69	3.42	3.72	3.84	3.76
α-D-Glc*p*-(1→6)- (**D**)	4.984	3.54	3.74	3.44	3.73	3.85	3.78
7							
-(1→4)-α-D-Glc*p* (**Rα**)	5.230<br>92.7	3.57<br>72.2	3.96<br>74.0	3.61<br>78.6	3.97<br>70.9	3.88<br>61.3	3.83
-(1→4)-α-D-Glc*p* (**Rβ**)	4.656<br>96.6	3..27<br>74.8	3.77<br>76.9	3.61<br>78.6	3.62<br>75.5	3.95<br>61.7	3.81
-(1→4,6)-α-D-Glc*p*-(1→4)- (**A**)	5.349<br>100.6	3.64<br>72.4	3.97<br>74.0	3.67<br>78.5	4.03<br>71.1	3.87<br>68.1	3.95
-(1→4)-α-D-Glc*p*-(1→6)- (**B**)	4.977<br>99.4	3.59<br>72.1	4.01<br>74.2	3.66<br>78.4	3.86<br>71.2	3.89<br>61.3	3.83
α-D-Glc*p*-(1→4)- (**C/C'**)[b]	5.360/5.368<br>100.6	3.56/3.58<br>72.6	3.69<br>73.8	3.43/3.41<br>70.1	3.73<br>73.6	3.84<br>61.2	3.75

[a] In ppm relative to the signal of internal acetone (δ 2.225 for ^{1}H and δ 31.07 for ^{13}C).
[b] Residue C and C' can be interchanged.
